# A nuclear targeting system in *Plasmodium falciparum*

**DOI:** 10.1186/1475-2875-9-126

**Published:** 2010-05-14

**Authors:** Kanjana Wittayacom, Chairat Uthaipibull, Krittikorn Kumpornsin, Ruchanok Tinikul, Theerarat Kochakarn, Pucharee Songprakhon, Thanat Chookajorn

**Affiliations:** 1 Department of Biochemistry, Faculty of Science, Mahidol University, Bangkok 10400, Thailand; 2 Protein-Ligand Engineering and Molecular Biology Laboratory, National Center for Genetic Engineering and Biotechnology (BIOTEC), Science Park, Pathumthani, Thailand; 3 Division of Medical Molecular Biology, Department of Research and Development, Faculty of Medicine Siriraj Hospital, Mahidol University, Bangkok 10400, Thailand

## Abstract

**Background:**

The distinct differences in gene control mechanisms acting in the nucleus between *Plasmodium falciparum *and the human host could lead to new potential drug targets for anti-malarial development. New molecular toolkits are required for dissecting molecular machineries in the *P. falciparum *nucleus. One valuable tool commonly used in model organisms is protein targeting to specific sub-cellular locations. Targeting proteins to specified locations allows labeling of organelles for microscopy, or testing of how the protein of interest modulates organelle function. In recent years, this approach has been developed for various malaria organelles, such as the mitochondrion and the apicoplast. A tool for targeting a protein of choice to the *P. falciparum *nucleus using an exogenous nuclear localization sequence is reported here.

**Methods:**

To develop a nuclear targeting system, a putative nuclear localization sequence was fused with green fluorescent protein (GFP). The nuclear localization sequence from the yeast transcription factor Gal4 was chosen because of its well-defined nuclear localization signal. A series of truncated Gal4 constructs was also created to narrow down the nuclear localization sequence necessary for *P. falciparum *nuclear import. Transfected parasites were analysed by fluorescent and laser-scanning confocal microscopy.

**Results:**

The nuclear localization sequence of Gal4 is functional in *P. falciparum*. It effectively transported GFP into the nucleus, and the first 74 amino acid residues were sufficient for nuclear localization.

**Conclusions:**

The Gal4 fusion technique enables specific transport of a protein of choice into the *P. falciparum *nucleus, and thus provides a tool for labeling nuclei without using DNA-staining dyes. The finding also indicates similarities between the nuclear transport mechanisms of yeast and *P. falciparum*. Since the nuclear transport system has been thoroughly studied in yeast, this could give clues to research on the same mechanism in *P. falciparum*.

## Background

*Plasmodium falciparum *is responsible for the deadliest form of human malaria. The underlying gene control mechanisms have been an active area of research for years owing to the potential for drug and vaccine development [1]. The parasite has a membrane-bound nucleus with a haploid genome of 23-Megabases encoded in 14 chromosomes [2]. The parasite undergoes mitotic division in red blood cells to generate 8-24 new nuclei in approximately 48 hours. Its chromosomes contain telomeric and putative centromeric regions [3-5]. Despite the paucity of *P. falciparum *transcription factors with clear homologues in other species [6], stage-specific gene expression patterns were observed by microarray studies suggestive of transcriptional control [7,8]. The role of epigenetic gene regulation in the parasite is becoming clearer, with revised gene annotation revealing components of histone-modifying enzymes and modified histone readers [9]. Furthermore, nuclear compartmentalization has been suggested to play an important role in gene regulation by forming transcriptionally active and silenced areas in the nucleus [10]. Gene control mechanisms in malaria parasites are a fascinating subject, but there are few molecular toolkits available to study the nucleus of *P. falciparum*. The ability to specifically transport a protein of choice to the nucleus is a valuable tool in many model organisms. This technique could be used to dissect the molecular basis of *P. falciparum *nuclear function by targeting of exogenous proteins into the nucleus.

Malaria parasite uses short peptide sequences to determine the destination of newly-synthesized proteins. For example, the PEXEL/VTS sequence is responsible for targeting malarial proteins to the red blood cell membrane [11,12]. Proteins are also transported into the apicoplast using a bipartite signal sequence to facilitate apicoplast targeting and membrane translocation [13,14]. These findings open new opportunities for developing molecular toolkits for targeting a protein of choice to various malaria organelles. An example of this organelle targeting approach is the use of Pfhsp60 N-terminal sequence to send proteins to the mitochondrion [15]. To address the lack of a nuclear targeting technique in *P. falciparum*, a similar approach was adopted by utilizing the yeast transcription factor Gal4 as a nuclear carrier protein. The Gal4 protein (Gal4p) is one of the first well-studied DNA-binding transcription factors in the budding yeast *Saccharomyces cerevisiae *[16]. The protein is actively imported into the yeast nucleus and binds to *GAL *upstream activation sequence [17,18]. A nuclear localization signal (NLS) at its N-terminus is a protein region recognized by the yeast nuclear transport machinery, and is necessary for targeting Gal4p into the nucleus [16]. Since its nuclear localization sequence has been accurately delimited [16], the potential use of Gal4p as a nuclear targeting system in *P. falciparum *was tested. Here, the nuclear localization signal of Gal4p was shown to be compatible with *P. falciparum *and can effectively transport a protein of choice into the nucleus. Truncated versions of Gal4p were analysed by confocal microscopy to narrow down the minimal nuclear localization region functioning in the malaria parasite. The Gal4-nuclear targeting system was also shown to be compatible with different *P. falciparum *strains.

## Methods

### Plasmid construction

**Figure 1:**
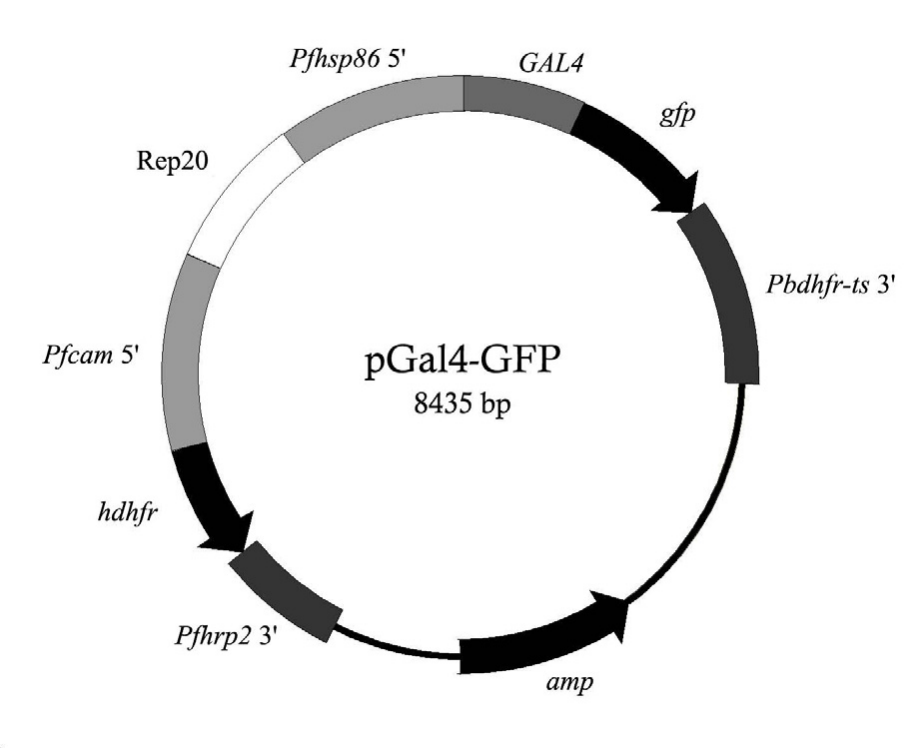
**Gal4 constructs for transporting GFP in *P. falciparum***. The pGal4-GFP construct has the first 147 residues of Gal4p fused to GFP. Expression of Gal4-GFP was controlled by the 5" and 3" flanking sequences of *Pfhsp86 *and *Pbdhfr-ts*, respectively. The selection marker was human *dihydrofolate reductase *(*hdhfr*), which allows selection by WR99210. A pGal4-GFP construct was divided further into pGal4(1-74)-GFP and pGal4(75-147)-GFP to narrow down the nuclear localization sequence. Rep20 was used to promote plasmid maintenance [26].

The N-terminal region (1-147 amino acid residues) of *S. cerevisiae GAL4 *from pGBKT7 (Invitrogen) was cloned into a modified pSSPF2/PfHsp60-GFP vector [15] to replace the PfHsp60 mitochondrial targeting sequence. The modified pSSPF2/PfHsp60-GFP vector has a linker (5'-CCTAGCGCTAGCAAATTAGGTACCTCAAGAGCAACAAATAATACTAGG-3') inserted into the *Avr*II cut site of the original pSSPF2/PfHsp60-GFP vector (a gift from Shigeharu Sato, National Institute of Medical Research, U.K). The *PfHsp60 *fragment was replaced by PCR-amplified *GAL4 *using *Bgl*II and *Kpn*I restriction sites (Figure 1). The construct was confirmed by direct sequencing and named pGal4-GFP. The N-terminal region of Gal4p was further truncated using the same cloning strategy to generate the pGal4(1-74)-GFP and pGal4(75-147)-GFP constructs which contain amino acid residues 1-74 and 75-147 of Gal4p, respectively. Large-scale plasmid preparation for transfection experiments was conducted according to the method described previously [19].

### Parasite culture and transfection

*Plasmodium falciparum *parasite strains 3D7 and K1CB1 used in this study were routinely maintained in fresh human red blood cells at 5% haematocrit in RPMI-1640 medium containing 10% human serum, 0.2% sodium bicarbonate and 40 mg/L gentamicin (complete RPMI medium) under 5% CO_2_, 1% O_2 _and 94% N_2 _atmosphere as described previously [20]. Transfection of the parasites was performed using a protocol described in [21]. In brief, transfection plasmids were introduced into ring-stage parasites by electroporation at 0.310 kV and 950 μF using a Biorad electroporator. Electroporated parasites were immediately transferred to complete RPMI medium and maintained as described above. Transfectants were obtained by selection with 3 nM WR99210 and were maintained in the complete RPMI medium containing the drug for the whole period of the study.

### Fluorescent and confocal microscopy

Localization of Gal4-GFP fusion protein in the transfected parasites was observed using an Olympus BX51 fluorescent microscope equipped with an Olympus DP71 microscope digital camera. GFP localization was also examined using a Zeiss LSM510 Meta laser-scanning confocal microscope. Images were analysed by DP Manager (version 3.1.1) equipped with LSM (release 3.2) software.

## Results

### Nuclear targeting system in *P. falciparum*

Yeast nuclear transcription factor Gal4p contains a nuclear localization sequence within its first 147 amino acid residues [18]. Its nuclear localization sequence can also function in other model organisms [22], making it a promising candidate for developing a nuclear targeting system in *P. falciparum*. To test this possibility, residues 1-147 of Gal4p were fused to the N-terminus of the GFP protein marker and expressed under the control of 5' and 3' flanking sequences of *P. falciparum heat shock protein 86 *(*Pfhsp86 5*') and *Plasmodium berghei dihydrofolate reductase-thymidylate synthase *(*Pbdhfr-ts 3*'), respectively. Transfection and drug selection by WR99210 were performed in the *P. falciparum *3D7 and K1CB1 strains.

**Figure 2:**
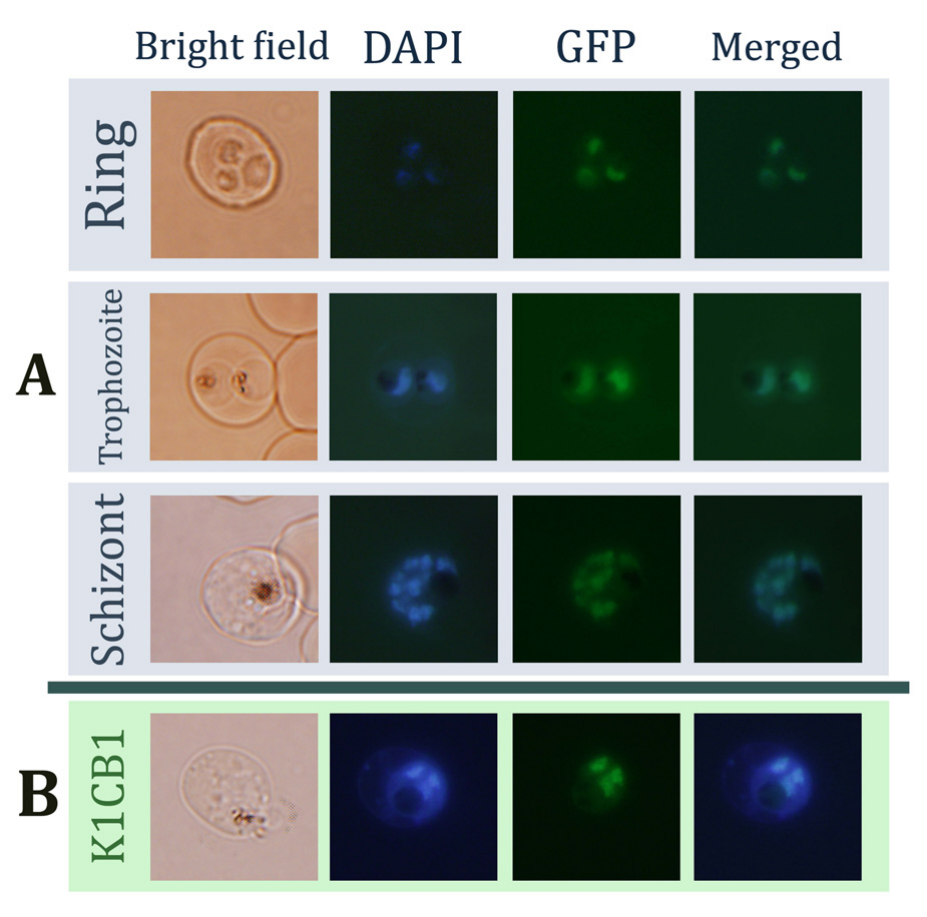
**Nuclear transport by Gal4p in *P. falciparum***. A) *P. falciparum *3D7 strain carrying the pGal4-GFP construct was studied by fluorescent microscopy. Three erythrocytic stages of *P. falciparum*, ring, trophozoite and schizont, are shown in four different panels of bright field, DAPI, GFP and DAPI merged with GFP. B) a similar experiment was performed with the K1CB1 strain. A trophozoite-stage K1CB1 parasite is shown here.

The transfected parasites were stained with DAPI to locate the nucleus. Gal4-GFP co-localized with the DAPI signal from the nucleus (Figure 2A). This finding hints at an effective nuclear transport mechanism in *P. falciparum*, as most of the Gal4-GFP signals were found in the nucleus. The GFP signal was found as early as in the ring stages and became more intense in the trophozoite and schizont stages (Figure 2A). The GFP signal from every erythrocytic stage was co-localized with the DAPI signal confirming the existence of Gal4-GFP in the nucleus (Figure 2A). GFP protein without a nuclear localization sequence from Gal4p could not be localized specifically to the *P. falciparum *nucleus. Hence, the Gal4-nuclear targeting system can be used to transport a protein of choice into the nucleus of *P. falciparum*. It also indicates that *P. falciparum *has a nuclear transport system that recognizes a nuclear localization signal of *S. cerevisiae*. It is worth noting that the *Pfhsp86 *promoter was found to have peak expression during the late-ring and trophozoite stages [8], which coincides with the strong GFP signal in the trophozoite stage.

To test whether the nuclear-targeting approach can work in another strain of *P. falciparum*, the pGal4-GFP construct was transfected into the K1CB1 line. The clear co-localization pattern of the DAPI and GFP signals indicated that this approach is likely to be functional in different *P. falciparum *cultured lines (Figure 2B).

### Locating nuclear localization signal

**Figure 3:**
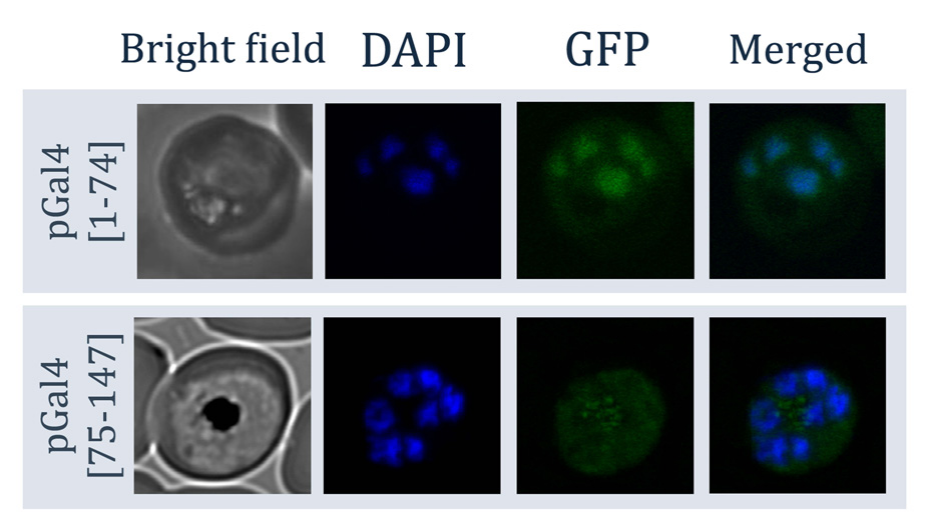
**Confocal microscopy of Gal4p fragments in *P. falciparum***. Two truncated forms of Gal4-GFP were constructed and labeled as pGal4(1-74)-GFP and pGal4(75-147)-GFP. Bright field, DAPI, GFP and DAPI merged with GFP images were taken by a laser-scanning confocal microscope from late-stage parasites. The first 74 residues of Gal4p were sufficient for transporting GFP into the nucleus as shown by confocal microscopy.

In order to reduce the complexity of the nuclear targeting system and minimize the risk of confounding functions caused by the Gal4 protein itself, it is necessary to narrow down and determine the minimal region necessary for nuclear transport. It was shown in yeast that only the first 74 residues of Gal4p are sufficient for nuclear transport [18]. To test if this is also the case with *P. falciparum*, the first 147 residues of Gal4p were divided into two sections. Residues 1-74 and 75-147 of Gal4p were fused with GFP to create pGal4(1-74)-GFP and pGal4(75-147)-GFP vectors, respectively. Laser-scanning confocal microscopy was employed to study the distribution of tagged GFP in detail. The result showed that only Gal4(1-74)-GFP fusion protein can enter the nucleus of *P. falciparum *(Figure 3). The GFP signal from Gal4(75-147)-GFP fusion protein was not specifically co-localized with the DAPI signal, indicating that the nuclear localization sequence from Gal4p is important for nuclear transport, and GFP alone is not sufficient for nuclear transport (Figure 3). The Gal4(75-174) protein also formed punctuated foci, which could be malaria food vacuoles (Figure 3). The images from confocal microscopy allow us to see even distribution of the GFP signal in the nucleus. Constructs shorter than 74 amino acid residues were not tested as these sequences function inconsistently for nuclear transport in *S. cerevisiae *[18], which would defeat the purpose of developing an efficient and reproducible nuclear targeting system for *P. falciparum*.

## Discussion

After invading the red cell, malaria parasites form an elaborate network of membranous structures and organelles. The malaria parasite must have a well-designed targeting system to make each organelle work together in harmony. Understanding the organelle targeting system provides an approach to experimentally manipulate the parasite. Here, the parasite also seems to rely on a similar mechanism to transport proteins to the nucleus. However, a universal peptide signal for nuclear transport is not as apparent as the transport signals of other organelles, even in well-studied model organisms. The lack of canonical nuclear transport signals could result from a plethora of adaptor proteins, or the availability of a piggyback mechanism.

In general, proteins are imported into the nucleus via the nuclear pore complex (NPC) which is a multi-protein channel spanning the nuclear membrane [23]. Its transport mechanism is tightly controlled by a small GTPase protein family [24]. Many putative orthologues of the NPC components have been identified in *P. falciparum *and other Apicomplexan species by sequence comparison [24]. The majority of the NPC proteins, such as RAN GTPase and importin exist in *P. falciparum *[24], which could explain the effective transport of yeast Gal4-GFP into the nucleus. The nuclear import activity of Gal4p in yeast was proposed to be mediated by importin β, which has a homologue in *P. falciparum *[24,25]. The fact that Gal4p can be used in an Apicomplexan species like *P. falciparum *also suggests the possibility of using this protein for the nuclear transport application of other parasitic protozoa.

An efficient nuclear targeting system has several potential uses in malaria research. For example, a study to investigate the activity of an enzyme that activates a compound of choice and causes a specific reaction within the nucleus could be performed. The nuclear tagging system can also be developed as an assay for screening of chemical compounds modulating nuclear transport in the parasite, for identifying the NPC components in *P. falciparum*, and for tracking organelle biogenesis. This Gal4 nuclear targeting system provides a new addition to the molecular toolkits for tracking *P. falciparum *organelles.

## Conclusion

A nuclear targeting technique was developed for effectively sending a protein of choice to the nucleus of *P. falciparum *by using the nuclear localization signal of yeast Gal4p. The finding indicated similarities between the nuclear import machineries of yeast and *P. falciparum*.

## Competing interests

The authors declare that they have no competing interests.

## Authors' contributions

KW and TC performed molecular cloning work. CU created transfected lines. RT and KK confirmed the transfection. KW and CU studied the parasites by fluorescent microscopy. KW and PS studied the parasites by confocal microscopy. TK performed image analysis. CU and TC prepared the manuscript. TC conceived the project. All authors read and approved the final manuscript.
